# Visualizing dynamic capabilities as adaptive capacity for municipal water governance

**DOI:** 10.1007/s11625-016-0408-y

**Published:** 2016-11-04

**Authors:** Jeffrey M. Widener, Travis J. Gliedt, Preston Hartman

**Affiliations:** University of Oklahoma, Norman, OK USA

**Keywords:** Adaptive capacity, Digitalization and visualizations, Environmental change, GIS, Oklahoma, Sustainable water governance

## Abstract

This study seeks to expand empirical research on how municipalities have adapted and innovated (or not) their water systems as a result of climate change. We analyze characteristics of water governance at the municipal scale in Oklahoma, USA. ArcMap 10.3 was used to build a qualitative geographic information system (GIS) based on fieldwork, including interviews and site-observations, to compare dynamic capabilities that lead to innovation in 38 cities in the state. The GIS enables visualization of our digitalized research to understand the interconnections between drivers of innovativeness—the combination of dynamic capabilities and innovation rates—and state of water resource infrastructure in place specific and regional planning contexts. In particular, the GIS takes into consideration income level, the influence of state-level water policy (Water for 2060 Act), water manager certification levels, population, dynamic capabilities, and perceptions of risk and vulnerability to water system change. Digitizing this information provides a diverging perspective on the historical lack of innovation in the public sector, as different socio-cultural, socio-economic, and socio-political contexts occur throughout Oklahoma, a state notorious for its oil centered economy and its climate change deniers. The findings suggest that innovativeness is directly related to dynamic capabilities and indirectly related to population size, income level, and the educational backgrounds of water decision-makers. The visualizations also show that some cities have surplus capacity for adaptation, while others were able to more efficiently turn capacity into water management innovations. Seeing representations of water governance success and failure in communities affords the opportunity to educate citizens and decision-makers to adapt water infrastructures to the effects of climate change, showcasing the utility of digitalization in a quest for sustainable solutions.

## Introduction

The conflict over control, management, and allocation of water resources is a centuries old global phenomenon. A trend that has gripped the attention of organizations, politicians, researchers, and people experiencing the effects of climate change is how water governance relates to sustainability and development (Castro 2007). The Global Water Partnership (GWP) defines water governance as the “range of political, social, economic and administrative systems that are in place in a particular country to manage water and deliver services” (GWP 2015). Besides social, economic, and political controls, this definition also sheds light on two other controls imperative to water governance; scale and boundaries (Davidson and de Loë 2014; Mitchell 2005). Even in micro-scale studies, research typically suggests that the outcomes of water governance are global in scope and should be managed as a collective resource so that all of humanity and Earth’s ecosystems have equity in the system (Huntjens et al. 2011). As such, analyzing how water is managed at micro-scales is important to build an understanding of socio-political conditions, uncertainties, and interdependencies (Armitage 2005). To be sure, the GWP maintains that “how a country manages its water resources determines the health of its people, the success of its economy, the sustainability of its natural environment, and its relations with the neighbors.” But how a country or state manages its water greatly depends on its varying local geographies, how municipalities manage their systems, and how communities adapt to multiple uncertainties, including drought.

**Fig. 1:**
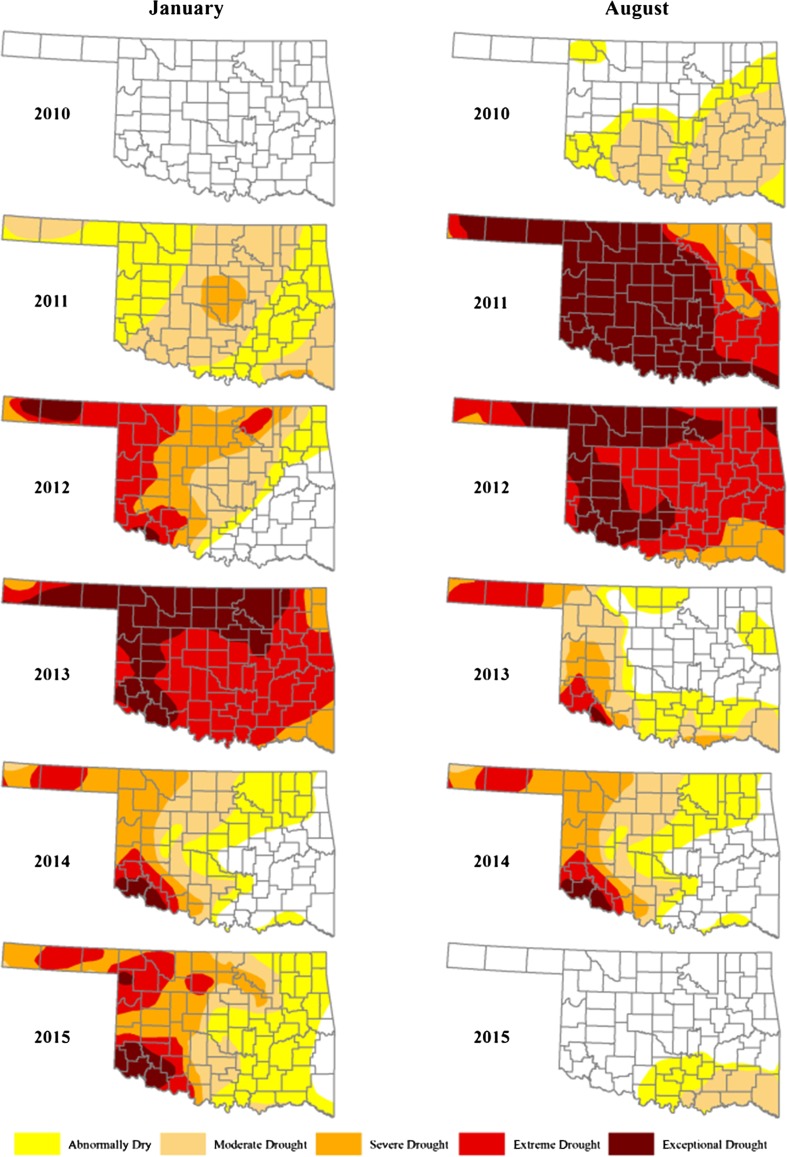
US Drought Monitor, Oklahoma 2010–2015. The maps highlight the severity and longevity of the drought and how it changed between 2010 and 2015 in Oklahoma’s driest months (January and August). The drought influenced adaptive water governance in municipalities across the state (NDMC 2016; USCB 2015)

This paper focuses on 38 municipal water systems in the state of Oklahoma, USA, and how each of their water managers applied innovativeness—the combination of dynamic capabilities relative to innovations—to their water systems as a result of climate change. The study took place in 2014, during the peak of one of the worst droughts that Oklahoma has experienced (Fig. 1). Indeed, different areas of the state experienced drought differently. Water managers’ responses and actions, their governance, to deal with the effects ranged from the governor’s spiritual request for “prayers for rain,” to Oklahoma City—the state’s largest metropolitan area in terms of scale and population—calling water from distant reservoirs that it owns, to cities in the southwest portion of the state, the hardest hit region, adopting innovative measures to combat the water deficit. An example includes one municipality’s implementation of “Stage 3 Water Restrictions,” which limited residents to handheld watering; no residential car washes, only commercial; no filling swimming pools; and for commercial kitchens, no “thawing food with water” or “cleaning kitchens and food handling areas with spray hoses” (Allen 2015; Layden 2015; Potter 2014; Talley 2013). To this day, many of these ideas and restrictions remain in place because of the variability and uncertainty in climate change.

One way for water managers to proactively engage uncertainty is through the digitalization and sharing of information. Seele (2016) maintains that digitizing disparate data enable stakeholders the opportunity to “rigorously observe and compare sustainability performance” (p. 849). Indeed, digitalizing sustainability performance allows us to visualize patterns, processes, and results that, in turn, can lead to better planning and even resiliency. Water managers, in particular, can benefit from data digitalization to achieve informed governance of water resources and improve delivery to consumers (Laine 2013). Doing so would render possibilities of achieving sustainability. This is, perhaps, especially true during times of drought, as impacts from drought episodes occur over longer periods of time rather than in short-term intervals and have long lasting impacts. Data digitalization, as it pertains to effective water governance, also affords water managers the potential to share information and manage water resources collaboratively rather than individually.

Digitizing data procured from our interviews and surveys with 38 water managers in Oklahoma enabled us to understand how precipitation changes relative to the long-term trend were associated with water system innovativeness. Our findings suggest that feeling, or experiencing, actual climate changes may influence greater water system innovativeness than population, municipal capital, and water managers’ certification/education level. To draw meaningful conclusions, we utilized other readily available data sources, including voting patterns, precipitation data, and income and population averages from census data. Each dataset was mapped in geographic information system (GIS) using ArcGIS software. Visualizing place specific components of water governance presented us with new interpretations of the variations and similarities that existed within our data. Furthermore, by visualizing these differences and similarities in 38 Oklahoma communities, the complexity of water decisions can be highlighted to help foster a more sustainable water future in accordance with the goals of the state’s Water for 2060 Act.

This study begins with a brief review of literature on water governance and its resilience characteristics, especially adaptive capacity and dynamic capabilities. Next, we discuss the methodologies used to conduct this study, including our digitalization and visualization approach. Then, we present our visualizations and highlight several important patterns with the help of the qualitative data. We conclude with how digitizing qualitative information assembled from interviews can help researchers spatialize their data to showcase water governance and sustainability in place specific contexts.

## Visualizing water governance

Understanding how municipal water systems and their managers are learning and adapting depends on sound and accessible data. Water systems represent a socio-technical system that includes “water, ecological and climatological interfaces, infrastructure, laws, consumer practices, and public and private-sector organizations” (Hornberger et al. 2015, p 4635). Therefore, data on water systems should contain a variety of factors, including climate predictions, organizational adaptability, water usage, population characteristics, education/training of water managers, economic data, and infrastructure details. Institutional actors could benefit from information systems that collect, analyze, and display sustainability and resilience characteristics of the water system management process, including elements of adaptive management, adaptive capacity, and dynamic capabilities.

### Adaptive water governance

One approach that institutions and communities adopt for water governance is adaptive management—the systematic process in which management continually improves its strategies based on what has been learned from previous management outcomes (Benson et al. 2015; Engle et al. 2011; Pahl-Wostl 2007; Pahl-Wostl et al. 2007). Adaptive management can lead to system change, including socio-political change that alters water usage and demand, physical infrastructure retrofits that make water supply and delivery more sustainable, and ecological forethought that considers the sustainability of the Earth for posterity (Pahl-Wostl 2007). Adaptive management is place and system dependent because each faces dissimilar uncertainties, including land use, population, economic activity, cultural factors, political circumstances, climatic conditions, and demand for services (Smit and Wandel 2006; Ivey et al. 2004). Instead, it is more the recognition of and willingness to experiment with management tactics to address uncertainties that characterizes adaptive water governance.

Oftentimes, the mentality is to do the bare minimum to carry out the adaptive management, thereby depriving the management approach of teeth. Adaptive management involves:

exploring alternative ways to meet management objectives, predicting the outcomes of alternatives based on the current state of knowledge, implementing one or more of the alternatives, monitoring to learn about the impacts of management actions, and then using the results to update knowledge and adjust management actions. Adaptive management is rooted in the process of learning and adapting, through partnerships of managers, scientists, and other stakeholders who learn together how to create and maintain sustainable resource systems (according to Williams et al. 2009 p. 1).

Organizations and communities cannot afford to remain risk adverse due to path dependence. Engle et al. (2011) argued that any “management that treats different aspects of water, e.g., hydrological, ecological, and socio-economic, separately, ignores their inherent interdependency, possibly at the expense of long-term sustainability” (p. 1). Management routines and processes, then, must be dynamic and evolve alongside the regulatory, social, economic, and ecological changes (Gunderson and Light 2006; Dessai and Hulme 2007). Adaptive water management benefits from integrating scientific knowledge and local knowledge, and institutional mechanisms can help stakeholders to continually coproduce and interpret this knowledge (Simpson et al. 2015).

### Building adaptive capacity

Adopting adaptive management principles in water governance can lead to a higher degree of adaptive capacity in a system, be it ecological, socio-political, or socio-ecological, to “adapt to change and respond to disturbances” (Armitage 2005, 706). As Armitage (2005) noted, several authors have refined the concept to fit their research needs (Olsson et al. 2004; Smit and Wandel 2006; Walker et al. 2002), but the general idea is to manage common resources, such as water, through experiments, learning processes, and acting on that knowledge to “foster innovative solutions in complex social and ecological circumstances” (Armitage 2005, 703). The end goal is to become more resilient and reduce vulnerabilities (Folke et al. 2002).

### Institutional capacity

To manage a water system effectively and increase its adaptive capacity, institutional actors and water users must understand the issues that surround their current system and have the ability to predict how the system might be disturbed (DeOreo 2006; Lockwood et al. 2015). For instance, systems that are expected to experience increased growth should respond proactively to ensure adequate water supplies for residents and businesses. Raw water assets, whether it is purchased water, groundwater, or surface water, are vital to adaptive management and increasing capacity. The source of the water supply and demand forecast information may influence how local water managers interpret scientific information from higher governance bodies (Damanpour and Schneider 2009; Lemos 2008, 2015). While one study in Ontario, Canada, found that some watershed-level governance decision-makers perceived technical scientific knowledge to be more valid than local knowledge (Simpson et al. 2015), differences in the water decision-making context in Oklahoma (e.g., local distrust of government and state agencies) suggest a potentially different interpretation of the value of scientific versus local knowledge. This may also differ spatially and between urban and rural areas within Oklahoma, where different historical traditions characterize their connection to higher-level government agencies.

Many factors influence the ability of organizations to produce adaptive measures (Ivey et al. 2004). Organizational skills such as managerial competence, resources, infrastructure quality, risk aversion of employees, and formal institutions guide adaptive decision-making within organizations (Arnell and Delaney 2006; Lockwood et al. 2015). Boyle (2014) suggests that utilities should first address financial constraints by adjusting user rates that “reflect the incremental cost of producing another unit of water, taking into consideration long-term water resource capital costs and the rising costs associated with water shortages” (p. 4). Water infrastructure is also a crucial component affecting adaptive capacity, and a 2009 American Society of Civil Engineer study gave the USA a ‘D’ grade on water system infrastructure (Boyle 2014). Drinking water and waste water infrastructure once again received a ‘D’ in the 2013 report (American Society of Civil Engineers 2013). Solving this infrastructure deficit will require adaptive management and institutional innovativeness.

### Consumer capacity

Another vital aspect of water utility adaptive capacity is the degree to which a utility can influence consumer behavior (Arnell and Delaney 2006; Lockwood et al. 2015). Maintaining good relationships with customers and government officials is especially important in systems that seek authorization for new innovations that do not have regulatory support (Bulkeley and Betsill 2005). Without broad support from the public, necessary funding from such options as water rate increases may not be possible because utilities are subject to the approval of city councils. Implementing successful adaptation strategies involves coordination spanning multiple jurisdictions and departments, which makes communication and the allocation of responsibilities more difficult (Berkhout et al. 2006). Governments may initiate adaptive measures through regulations and mandates (Adger et al. 2005). To be successful, these adaptive policies must be based on trust, reciprocity, and the value of social networks (Lockwood et al. 2015).

### Infrastructure Capacity

Adger et al. (2005) argued that adaptations can be organized in three categories: reducing sensitivity, altering exposure, and increasing resilience to cope with changes. Increasing potable water reservoir capacity or drilling new water wells that provide reserve supply for emergency cases are examples of measures that reduce sensitivities. Altering exposure entails the investment in new or upgraded infrastructure that is more climate resistant, and increased resiliency of a system can occur through actions that enhance the general public’s comfort and security while also strengthening the community’s ability to withstand and recuperate against various stressors and losses. Resilience is also enhanced by incorporating flexibility of information collection and exchange, as well as flexibility of water storage and movement within the water system—all of which would benefit from digitalization and the sharing of information.

### Identifying and deploying capacity

The process of identifying and deploying various adaptations is complex and often constrained by institutional forces. Municipal adaptations are first spurred by assessing the nature and scale of system vulnerabilities (Bulkeley and Betsill 2005). Significant hurdles are apparent at this stage, the most notably being the lack of adequate data concerning local scale impacts as a result of climate change and current quality assessments of system infrastructure. Adaptation at the urban scale has largely been limited to knowledge accumulation and strategy development. Financial resources and the lack of power in decision-making processes by management are largely to blame for the inability to enact adaptations. Zimmerman and Faris (2011), for example, found that North American cities have largely remained stagnant in their pursuit of water governance that considers climate change adaptation and policy. Experiments that may prove beneficial to more widespread adoption of climate change adaptations occur merely as “interventions to try out new ideas and methods in the context of future uncertainties” (Broto and Bulkeley 2013, p 92). Adaptive capacity can be generated through effective management that places significant emphasis on learning (Pahl-Wostl 2007); at the same time, management processes and routines can become “more adaptive and flexible to make it operational under fast changing socio-economic boundary conditions and climate change” (Pahl-Wostl 2007, p 51).

Water governance can contribute to sustainability value creation for urban water systems (Marlow et al. 2013). This requires more significant changes than the current trend in water management of treating and delivering low cost water to consumers (Marlow et al. 2013). Faced with the uncertain impacts of climate change, past knowledge of urban water systems and water resources must coevolve with ongoing water system knowledge generation to become more dynamic (Kiparsky et al. 2012). Wiek and Larson (2012) maintain that sustainable water governance requires “coordinating all relevant actors in their water-related supply, delivery, use, and outflow activities in a way that ensures a sufficient and equitable level of social and economic welfare without compromising the viability and integrity of the supporting hydro-ecosystems in the long term” (p. 3156). Such an approach advocates for the use of sustainable yield as part of an adaptive capacity and learning-centered system.

### Measuring adaptive capacity through dynamic capabilities

One way to measure the extent to which water utilities are building adaptive capacity is to view managerial actions through a dynamic capabilities lens. Teece et al. (1997) defined dynamic capabilities as the “ability to integrate, build, and reconfigure internal and external competences to address rapidly changing environments” (p. 516). Innovativeness can be measured as an output of dynamic capabilities learning processes, and thus as a measure of how organizations build adaptive capacity and execute adaptive water management (Lieberherr and Truffer 2015). This approach can help fill the “serious knowledge gaps and the lack of a sound conceptual base to understand learning and change in multi-level governance regimes” (Pahl-Wostl 2009, p 355).

### Information systems, digitalization, and visualization of water governance

The digitalization of data opens new opportunities, particularly in the sustainability arena. Since the goal of adaptive water governance is system resilience, sharing what has been learned and what capabilities were beneficial can enhance other water utilities’ efforts of becoming more sustainable (Chen et al. 2008; Robinson et al. 2006). Digitalizing content makes things more transparent, and depending on the type of information system used, it can also lead to real-time education. Indeed, if water managing organizations established an information system for digitalizing material related to their adaptive capacity building techniques via their dynamic capabilities, they could improve their efficiency and ability to use the information to enhance system resilience, while establishing a potentially long lasting database for others to reference (Standing and Jackson 2007). A recent example is the creation of the New California Water Atlas (NCWA), which

tells the story of California water, makes data about water resources more open and transparent, and provides tools to activate effective citizen participants. NCWA…re-imagines the role of an Atlas in today’s real-time web and big data era. [NCWA will] provide users with an intuitive and smoothly functioning interface for understanding the state’s water system, which serves as a platform for sharing stories and data.

The builders of the atlas explain that “data about this complex [water] system is woefully unorganized, inconsistent, and difficult to navigate. Information is scattered across many sites, and users are confronted by multiple data formats with few options for automated retrieval” (The New California Water Atlas 2015). And herein lies the overarching problem: big data is of no use unless it is readily accessible and usable.

With advances in technology, researchers can now digitalize disparate forms of data. Digitalization as a process enables researchers to visualize, join, and represent disparate data to form new interpretations. Visualizing the qualitative data has enabled us to see how actors and infrastructures in Oklahoma compare to one other based on patterns of population, precipitation, and income, and local water governance factors such as training, partnerships, and knowledge of water infrastructure improvement grants.

## Digitalization and visualization techniques for modeling processes of water governance

**Fig. 2:**
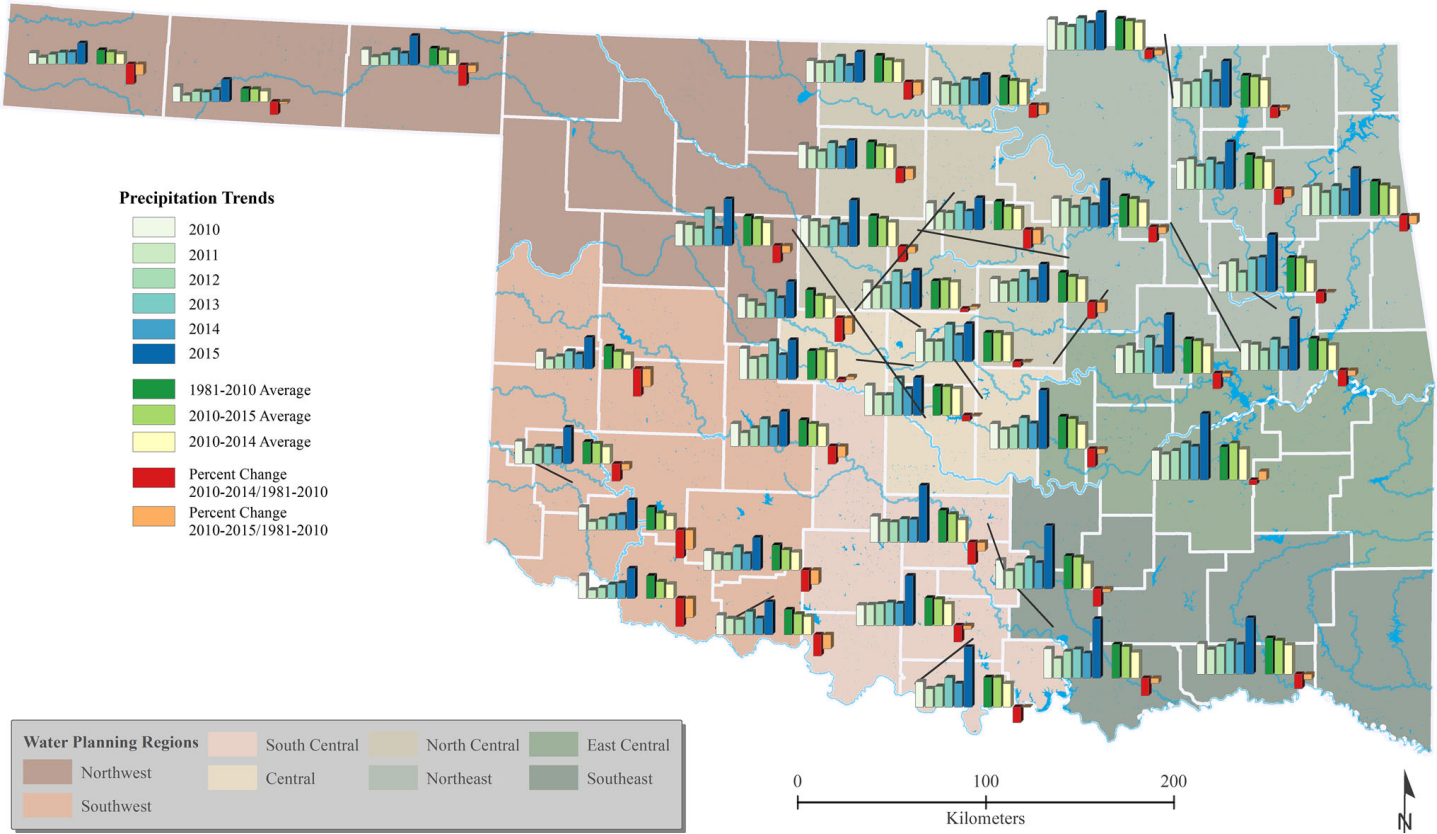
Precipitation Trends among Case Study Sites and Water Planning Regions Compared to Long Term Averages. The data represented in this map highlights the challenge for water governance in a state with extreme variability in precipitation patterns. The average precipitation received from 2010–2015, which includes a historic 2015 deluge, was less than the 30-year average. Where drought was most severe, more innovativeness took place (OCS 2015; USCB 2015)

Precipitation patterns in Oklahoma decrease as you move from east to west, varying from an annual average of over 50 inches in the east to as little as 17 inches of precipitation in the far northwestern Oklahoma panhandle (Fig. 2). It follows, then, that water governance occurs differently for different places in the state. The eight Water Planning Regions outlined by the Oklahoma Comprehensive Water Plan of 1995 serve as a framework for water governance to help understand and coordinate the management strategies of state and municipal water systems. These regions “exhibit common characteristics—such as homogeneity of climate, geography, hydrology, economics and demography—that meld them into functional planning units…each region is unique in its water resources and requirements” (OWRB 1995). Although a more recent Oklahoma Comprehensive Water Plan proposed modifying the Watershed Planning Regions in 2012 (OWRB 2012), it has not been approved (Layden 2013), and thus the 1995 plan outlines the official regions that were used in this study.

To understand multi-level differences in water governance, adaptive capacity, and innovativeness in Oklahoma, we utilized a mix of qualitative and quantitative information. Our primary data used in this study stem from semi-structured interviews conducted with municipal water decision-maker elites in each of the state’s eight water planning regions (Harvey 2011). We contacted 130 municipalities and rural water districts in Oklahoma and received a response rate of 29 percent, as 38 water decision-makers offered their time to complete, on average, a 75-mins interview. The semi-structured format allowed the interviewer to have more flexibility during the interview process (Leavy 2014). Interviews were in-person because interviewees give nonverbal cues, which the interviewer can observe and take note of (Cachia and Milward 2011). On a few occasions, the water decision-makers gave us a tour of their facility during the interview. Each interviewee consented to having the interview audio-recorded, which enabled better transcription and minimized loss of situational knowledge. We supplemented our interview data with independent information. This includes population and income characteristics data from the American Fact Finder website maintained by the United States Census Bureau and precipitation data made available through the Oklahoma Mesonet.

The incorporation of both qualitative and quantitative data in research has increased considerably over the last decade. Paschen and Ison (2014) maintain that:

narrative research offers a complementary approach to an improved, holistic, understanding of local socio-ecological systems. It yields potentially valuable data, including on local environmental knowledge(s), lay perceptions of climate change, and information on how socio-cultural and affective-emotive factors influence adaptive capacity that can significantly inform the design of local adaptation policies and public engagement strategies—features of systemic and adaptive governance (p. 1089).

We use GIS and cartographic visualizations to spatially represent the data of local water system knowledge. While several researchers use quantitative values in GIS, the use of qualitative data is comparatively more novel. Typically, when researchers refer to doing qualitative GIS, they tend to utilize a mixed-method approach that involves both quantitative and qualitative information (Knigge and Cope 2006; Kwan and Ding 2008). Johnson et al. (2007) call mixed method research the most “powerful” research paradigm that will “provide the most informative, complete, balanced, and useful research results” (p. 129).

Adding narrative knowledge to GIS and mapping that information in a critical, place-based context offers researchers the ability to communicate different ways of knowing through the use of cartographic representations (Bell and Reed 2004; Knigge and Cope 2009; Kwan and Ding 2008; Sheppard 2005). Mugerauer (2000) suggests that by developing qualitative GIS we can “access successfully one another’s life words rather than build enclaves through information technology” (p. 317). Pfeffer et al. (2011) add that qualitative GIS is a way for researchers to incorporate new socio-cultural knowledge that quantitative values and analysis do not always highlight. The digitalization of qualitative information and combining it with supplemental data in this capacity has the potential to close sustainability gaps in the quest for sustainable development.

With regards to digitalization and sustainability, a mix of qualitative and quantitative information in a GIS can not only help showcase local knowledge and innovativeness but it can also demonstrate trials and shortcomings of how these themes relate to other situated frameworks of knowing, such as the role of income, population, and climate dynamics in water governance (Worthington 2014). Our empirical case study on visualizing municipal water governance innovativeness and constraints in 38 Oklahoma water systems sheds light on these communities and provides a model for examining place-based efforts toward a more sustainable culture of global water governance.

## Results: visualizing dynamic capabilities in municipal water governance in Oklahoma

**Fig. 3:**
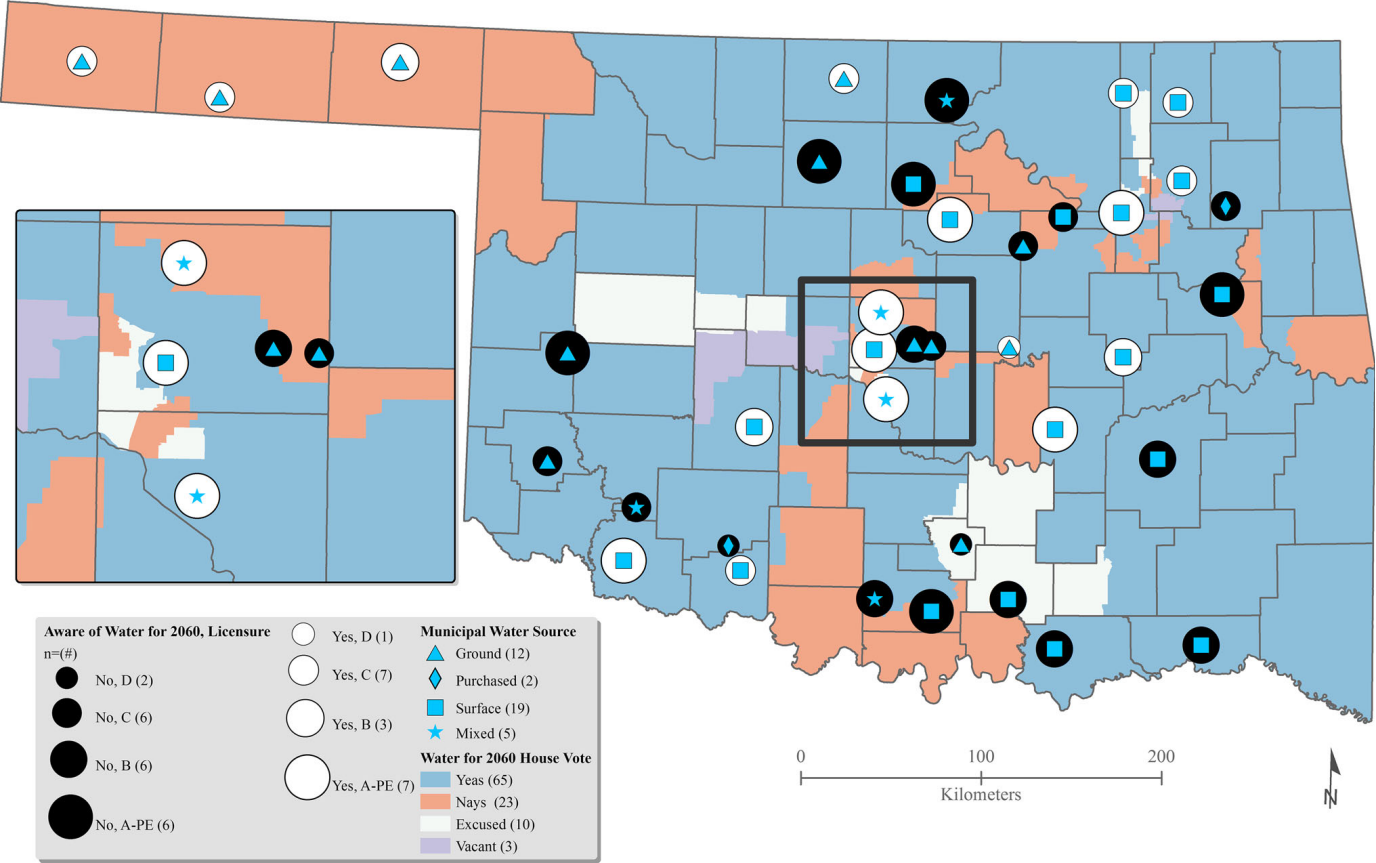
Knowledge of water for 2060: house votes and affiliation (House 2012; OSL 2015; USCB 2015)

**Fig. 4:**
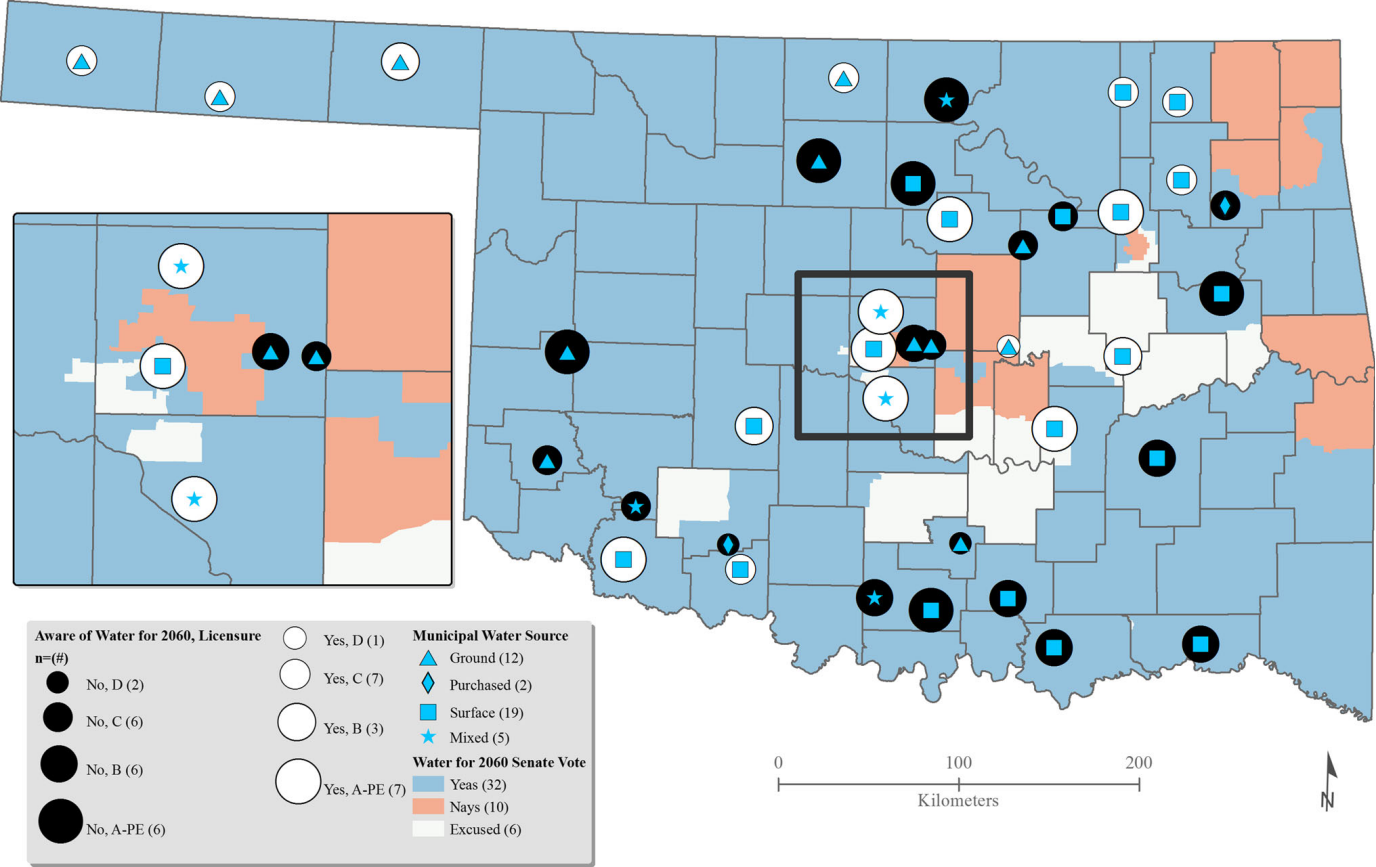
Knowledge of water for 2060: senate votes and affiliation (Senate 2012; OSL 2015; USCB 2015)

To comprehend how municipal water systems and their managers adapt water systems, a researcher should have a firm understanding on how politics and education might influence the decision-making process. The drought in Oklahoma was direst during 2011 and 2012. During this time, a statewide effort began to develop a bill known as the Water for 2060 Act. This voluntary water bill passed the Oklahoma governmental chambers in 2012, but when we digitized the voting process, and compared that with water managers’ knowledge of the bill, place-based patterns emerge. Figures 3 and 4 compare the municipal level water manager knowledge of the Water for 2060 Act and their knowledge of state-level water grants to the House/Senate party affiliation and House/Senate votes for Water for 2060. The pies on the map are sized based on the water manager certification (education) level, with D being the lowest level of certification and A-PE being the highest level of certification. The maps reveal a complex water governance landscape in the state, with awareness of the Water for 2060 and water grants highest in the Northwest and within a diagonal corridor running from the Southwest through the Central region to the Northeast. This is in contrast to the Southeast where few water managers were aware of Water for 2060 or grants. Perhaps, this is region’s lack of awareness which is attributable to its comparatively higher levels of precipitation. Understanding voting dynamics and water manager certification levels as well as their knowledge of Water for 2060 provides an important foundation for further analysis on overall water system innovations, adaptive capacity, and dynamic capabilities throughout the state.

**Fig. 5:**
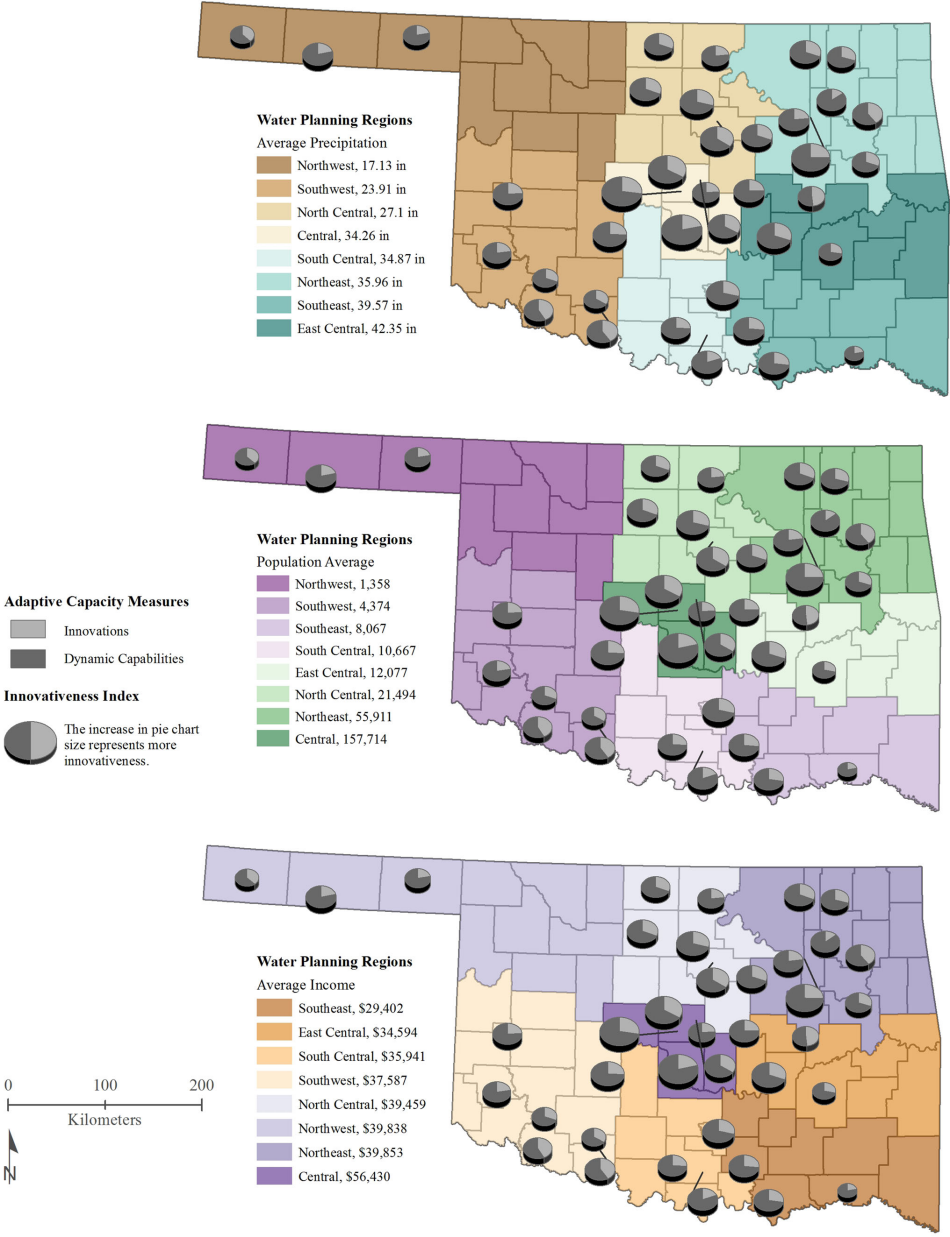
Adaptive capacity and innovativeness. The *pie charts* with greater portions of *dark gray* relative to *light gray* show surplus adaptive capacity. Innovativeness is visually represented by the size of the *symbol*, with larger ones having higher combined dynamic capabilities and innovations relative to the state average (USCB 2015)

Dynamic capabilities can provide adaptive capacity to water systems through their networking, flexibility, and learning attributes, leading to more innovativeness. It is therefore important to examine why some water systems have more dynamic capabilities relative to the number of innovations created. This is considered surplus adaptive capacity, which could be drawn upon to create water innovations in response to uncertain future precipitation patterns, or changes in population and corresponding water demand. The ratio of dynamic capabilities to innovations differs by water system. Water systems that have more than the state average of 2.6 dynamic capabilities per innovation created are considered to have surplus adaptive capacity (Fig. 5). There was no correlation between surplus adaptive capacity and innovativeness (*R* = 0.03; *p* = NS at *α* = 0.05). In other words, some water systems were highly innovative and highly efficient at turning capacity into innovations, while others were highly innovative despite having additional capacity for further innovation. The surplus capacity may exist either due to barriers or a lack of motivations.

Figure 5 also suggests that population and income are associated with innovativeness, while suggesting surplus adaptive capacity may be associated with precipitation levels in some regions (e.g., South Central, Southeast, and Southwest) but not others (e.g., Central, Northeast and the Northwest). For instance, 86% of the water systems in the South Central and Southeastern portion of the state have surplus adaptive capacity. This is in contrast to the 43% of water systems in the Southwest region and 37% of water systems in the Northeast region that have surplus adaptive capacity. The urban, high population centers were mixed, with Oklahoma City, Tulsa and Norman all having surplus adaptive capacity, while Stillwater, Edmond and Enid did not. It is important to note that the water systems facing frequent and intense droughts (Northwest and Southwest) were not the most innovative. This highlights the ongoing challenge for governing water system change within the state of Oklahoma in the face of climate induced pressures, as state-level resources and capabilities will need to be continually funneled into those areas.

Water utilities that were more innovative than the state average were more likely to have higher average incomes, populations, and water manager license certifications than utilities that were less innovative than the state average. On the other hand, innovativeness did not relate to knowledge of the Water for 2060 Act. In fact, nearly two thirds of water utilities that had low levels of innovation did have knowledge of Water for 2060. So knowing about the act does not appear to influence more innovativeness in the water system.

**Fig. 6:**
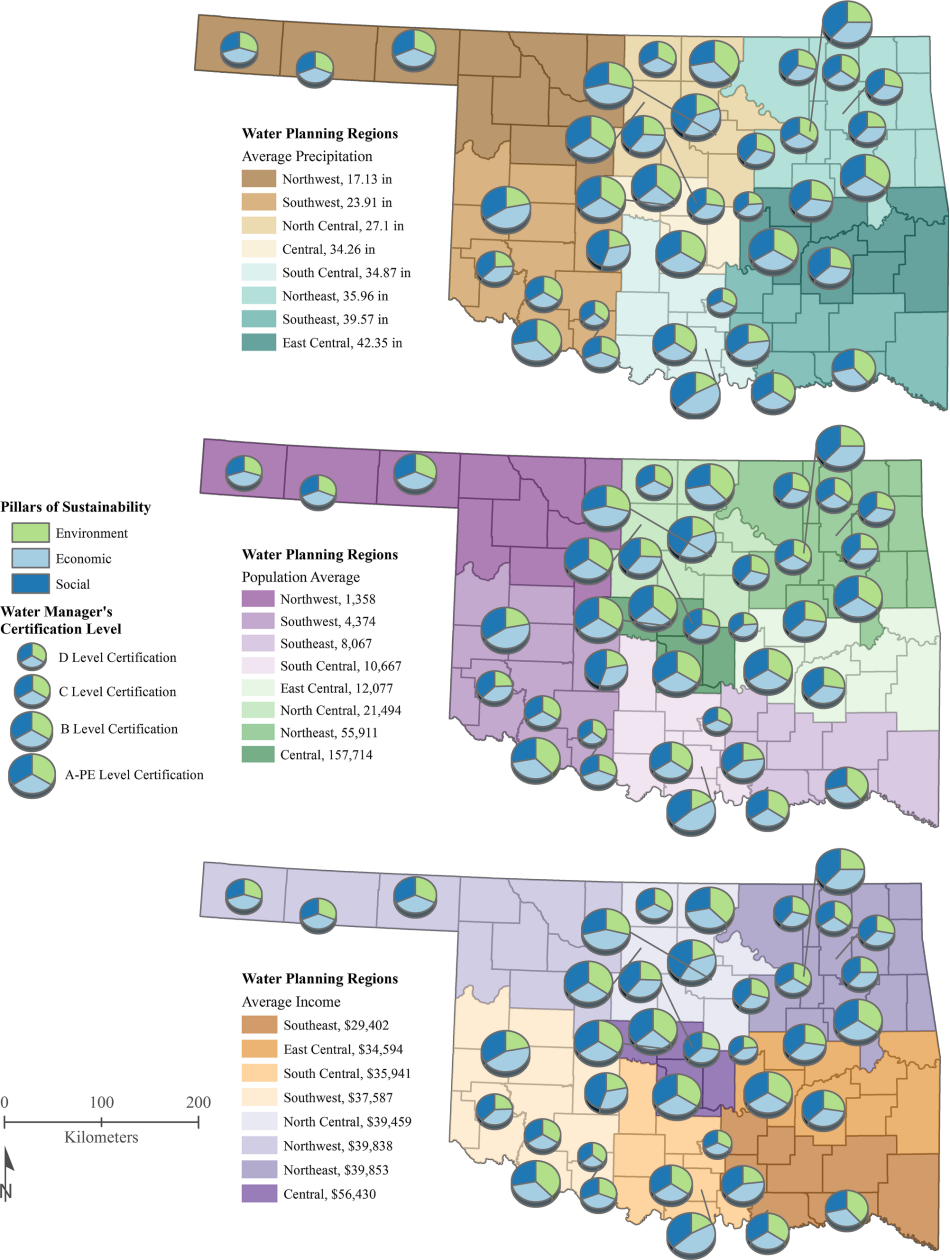
Motivations for water system innovation grouped by the pillars of sustainability: a comparison to water manager certification level relative to precipitation, population, and income. The environmental pillar includes promoting environmental sustainability and future changes to climate. The social pillar includes compliance with regulations, success of other water utilities, adequate water supply for the community, and adequate water supply for your kid’s generation. The economic pillar includes company reputation, risk management, internal economic reasons, availability of funds, and spurring local economic growth (USCB 2015)

**Fig. 7:**
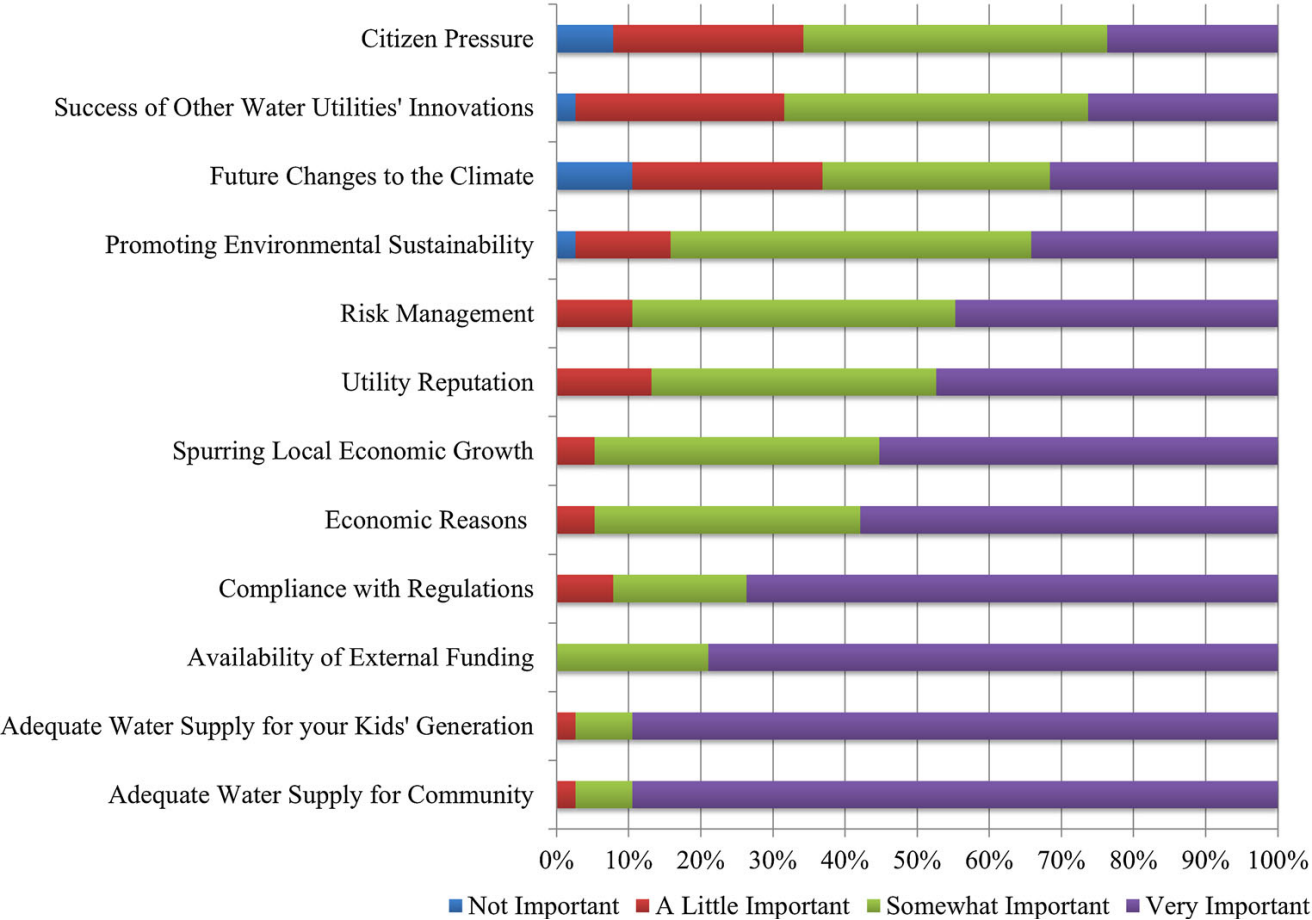
Motivations for water system innovation (*n* = 38)

Table 1 displays the importance of utilizing qualitative information to gauge various motivations for water system innovation. In Fig. 6, we mapped those motivations into the three pillars of sustainability to visualize place-based differences per water system. The most important factors to most water system managers were social and economic focused. It is interesting to note that the least important factor was citizen pressure despite these being public utilities that in theory all respond to taxpayers. The composition of the pie charts was normalized to visualize the relative importance of each of the three pillars of sustainability to water system innovation. The size of the pie represents the water manager certification level for that water system. Figure 7 showcases the info graphic adaptability benefit of having digitalized data, as the same information that Table 1 and Fig. 6 display is in Fig. 7.

**Table 1:** We asked each water manager a series of 24 questions

Example Interview qüuestion How important are the following items to creating new innovations	Coded for sustainability pillar	Counted motivation: very important	Counted motivation: somewhat important	Counted motivation: a little important	Counted motivation: not important
Economic reasons (cost savings, etc.)	Economic	22	14	2	0
Availability of funding (grants, etc.)	Economic	30	8	0	0
Spurring economic growth	Economic	34	3	1	0
Promoting environmental sustainability	Environmental	13	19	5	1
Future climate changes	Environmental	12	12	10	4
Adequate water supply for posterity	Environmental	34	3	1	0
Compliance with regulations	Social	28	7	3	0
Company reputation	Social	18	15	5	0
Risk management	Social	17	17	4	0
Success of other water utilities’ innovations/programs	Social	10	16	11	1
Stakeholder pressure	Social	9	16	10	3

This table presents an example of a Likert scale question for which each water manager ranked the motivation for creating new water system innovations as part of their water governance process. We also show how we grouped their responses into the commonly used pillars of sustainability: economic, environmental, and social—and the count for each level of motivation. This mixed method research process allowed us to map the motivations for each place in Fig. 6. Indeed, digitalizing this data makes for effective functionality for visualization purposes, as a variety of infographics can be created off the same data as shown in Fig. 7

## Discussion

We would like to encourage readers to return to Fig. 2. Over the past six years, Oklahoma, like many places around the world, has experienced the effects of climate change. Water systems in our 38 case study sites have been hard hit by a drying trend with lower amounts of precipitation than the 30-year average. Some systems have begun to instigate significant changes to water supply and demand management, while others demonstrated vastly lower levels of innovativeness. These differences can be attributed to resource-based dynamic capabilities, which were related to population, income and water decision-maker characteristics in each system.

This study has demonstrated how digitizing qualitative information assembled from interviews can help researchers spatialize their data to showcase water governance and sustainability in place specific contexts. While correlations existed between many of the important variables, the qualitative GIS analysis shows that the water governance landscape in Oklahoma is far more complex. Variation in socio-ecological and municipal governance characteristics suggests that a state-level water policy must be adaptable and customizable to encourage water system innovations within each region.

The identification of surplus adaptive capacity implies that some water systems have the potential to change given the right set of future characteristics. While previous studies pointed out the difficulty in identifying and measuring adaptive capacity in the absence of shocks (Engle 2011), the visualization analysis has shown that some regions do appear to have untapped adaptive capacity. For example, in the absence of drought or water shortages, water systems in the Southeast and South Central regions have made fewer changes relative to their capacity to change.

The innovativeness measure does not tell the whole story, however, as water constrained systems in the Southwest and Northwest regions were very efficient at turning capacity into actual innovations, yet produced fewer innovations relative to larger water systems. Interestingly, no regional patterns were evident regarding the composition of the importance of the three pillars of sustainability in relation to innovativeness. Rather, examples existed for water systems that were able to turn capacity into innovations when motivated more by economic, social, or environmental drivers relative to the other pillars. Despite the importance of water certification level to innovativeness, six water managers that had the highest A-PE certification level were unaware of the Water for 2060 Act. This suggests that municipal drivers for water system change may overshadow messages from the state concerning water conservation. This, coupled with the finding that knowledge of Water for 2060 did not correlate with innovativeness, implies that a new strategy is needed to both strengthen the Act as well as link its guiding structure to actual plans and funding options for addressing the water infrastructure deficit in municipalities.

Digitalization of sustainability information from research not only provides professionals and academics with the opportunity to analyze disparate data, digital information can enable governance regimes, in particular water managers, to create a check and balance type system. This system can proffer additional sustainable actions and resilient solutions for posterity. Some companies and institutions already employee digital strategies, including the Siemens Corporation, which argues that “through digitalization, systems and processes are enabled to provide valuable data which can then be analyzed and used to improve operating productivity, refine process flows, trigger preemptive maintenance, better align supply and demand, and generally enhance business effectiveness and efficiency (Siemens 2015, p 3). In Oklahoma, for example, the Oklahoma Gas & Electric company has employed smart metering, sending digital information on electric rates to users so that they can adjust their systems accordingly, and provide residents with a weekly energy summary that compares electric use to their neighbors and a flat efficient rate. There is no reason, then, why water managers could not develop similar means to help govern and monitor the world’s most invaluable resource.

Documenting success and failure is how we learn (Pahl-Wostl 2007). Water managers and consumers could benefit from such measures because they can jointly see through infographics, and quite possibly help manage water governance based on up-to-date information. Digitalization programs for water information in Oklahoma could fill knowledge gaps (Pahl-Wostl 2009), coordinate all actors in water management and water use (Wiek and Larson 2012), and guide consumers to make decisions based on rates, water supply, and user demand (Arnell and Delaney 2006; Lockwood et al. 2015). Building adaptive capacity through all the actors—institutions, consumers, and infrastructure—is an absolute must (Wiek and Larson 2012). Digitalization of data provides that monitoring and gathering nexus for building a platform through which citizens can begin to participate and effect change for more global adaptive, sustainable water governance.

As Worthington (2014) reminds us, however, creativity must “nudge an existing [system] in better directions” (p. 62). Nudging institutions in a digital direction could, in fact, lead to further adaptive capacity and dynamic capabilities because of the innovativeness involved in executing such an adaptive approach (Lieberherr and Truffer 2015). Information in databases would be more transparent, accessible, and archivable for future research and sustainability monitoring (Chen et al. 2008; Robinson et al. 2006; Standing and Jackson 2007). Indeed, the fast paced changing nature of technology and digital techniques will undoubtedly require its own set of adaptive capacity measures, but the rewards of digitalization exceed its initial shortcomings (Worthington 2014).

## Implications for digitalization on public policy

While the goal of this study was to detail how the digitalization and visualization of disparate data surrounding water governance in Oklahoma can lead to more transparency of place-based trends for research purposes, strong parallels can also be drawn for digitalizing data for creating effective public policy. Over the last few years, there has been particular interest among researchers on the process of integrated reporting (IR), led by the International Integrated Reporting Council (IIRC), and sustainability reporting (SR) as ways for institutions to voluntarily present how it achieved and or applied principles of sustainability into its practices (Perego et al. 2016). While most of the research on IR and SR focus on how businesses cooperate with detailing levels of their corporate responsibilities and financial performance (Adams et al. 2014), a business’s record of sustainability (Lozano et al. 2016), and how successes filter into innovations and system wide change, research on how the public sector use these frameworks is still emerging (Williams et al. 2011). Despite some scholars and professionals who have questioned motives and transparency in the IR and SR process (Jensen and Berg 2012; Flower 2014; Thomson 2015; Perego et al. 2016), the intentions and goals of both IR and SR are aspirational and provide frameworks from which governmental organizations, such as municipal water managers, could utilize to report sustainability progress (Adams 2015; Lozano et al. 2016; Mass et al. 2016).

The California Water Atlas as we described above could be viewed as an alternative IR and SR process, as the initiative provides an avenue for stakeholders to understand conditions and performance of water-related actions. Specifically, it serves as an example of a digital repository of data that professionals and the general public can utilize and view to effect change and create new efficient water policies based on past and present conditions for future consumption. Indeed, as we mentioned earlier, part of the problem with using and maintaining data in this manner is the gathering process, as data exists in different silos, is reported in different ways, and is often hard to interrelate (Garcia-Sanchez et al. 2013). As well, other data must be gathered through research processes such as those carried out in our project. Governmental institutions could, however, create streamlined questionnaires that decision-makers and managers complete each year with results being populated in databases that can be used for data interpretation, manipulation, and visualization. Seele (2016) outlines how businesses could do this thorough digitally unified reporting using the eXtensible Business Reporting Language (XBRL); public policy makers could modify such an approach to fit their needs.

Digitalizing IR and SR results can provide a foundation that institutions and stakeholders could adapt and build on. Benefits of having digitalized data related to any sustainable development initiative is the ability to manipulate data in purposeful ways, analyze that data, look for emergent patterns and/or trends, and potentially create infographics that explain performance (Otten et al. 2015). Policy makers can indeed benefit from such a process because they can build on successes or see how and why some initiatives failed in other locations to avoid repeating similar mistakes (Adams and Frost 2008; Barach and Lipshultz 2016). This, in turn, can lead to measures that could suture the “sustainability gap” (Lubin and Esty 2014; Seele 2016).

Technology and the digital world will continue to develop at a furious rate, further wrapping society into a data driven culture. Sustainability endeavors must keep pace or else we risk losing access to communities of innovators and global webs of knowledge that can lead and achieve a more sustainable public and environment for posterity. But as Seele (2015) warns, however, digitalizing efforts in the sustainability realm cannot become a “mere tick-box…by only fulfilling regulatory requirements” (p 11). Policy makers and stakeholders must work together to present a unified effort of digitalized sustainability information.
